# First identification of a recombinant form of hepatitis C virus in Austrian patients by full-genome next generation sequencing

**DOI:** 10.1371/journal.pone.0181273

**Published:** 2017-07-25

**Authors:** Evelyn Stelzl, Bernhard Haas, Bernd Bauer, Sherry Zhang, Ellen H. Fiss, Grantland Hillman, Aaron T. Hamilton, Rochak Mehta, Marintha L. Heil, Ed G. Marins, Brigitte I. Santner, Harald H. Kessler

**Affiliations:** 1 Institute of Hygiene, Microbiology and Environmental Medicine, Medical University of Graz, Graz, Austria; 2 Department of Internal Medicine, General Hospital Graz-West, Graz, Austria; 3 General Hospital Hörgas-Enzenbach, Gratwein, Austria; 4 Roche Molecular Diagnostics, Pleasanton, CA, United States of America; Medizinische Hochschule Hannover, GERMANY

## Abstract

Hepatitis C virus (HCV) intergenotypic recombinant forms have been reported for various HCV genotypes/subtypes in several countries worldwide. In a recent study, four patients living in Austria had been identified to be possibly infected with a recombinant HCV strain. To clarify results and determine the point of recombination, full-genome next-generation sequencing using the Illumina MiSeq v2 300 cycle kit (Illumina, San Diego, CA, USA) was performed in the present study. Samples of all of the patients contained the recombinant HCV strain 2k/1b. The point of recombination was found to be within the HCV NS2 gene between nucleotide positions 3189–3200 based on H77 numbering. While three of four patients were male and had migration background from Chechnya (n = 2) and Azerbaijan (n = 1), the forth patient was a female born in Austria. Three of the four patients including the female had intravenous drug abuse as a risk factor for HCV transmission. While sequencing techniques are limited to a few specialized laboratories, a genotyping assay that uses both ends of the HCV genome should be employed to identify patients infected with a recombinant HCV strain. The correct identification of recombinant strains also has an impact considering the tailored choice of anti-HCV treatment.

## Introduction

The hepatitis C virus (HCV) is a blood borne virus causing both acute and chronic hepatitis C. It is estimated that between 64 and 103 million people suffer from chronic hepatitis C worldwide [1]. HCV shows very high levels of genetic diversity having a major impact on the development of antiviral drugs, vaccines, and genotyping assays [2]. Currently, HCV is classified into 7 different genotypes and 67 subtypes [3]. Testing for the hepatitis C virus (HCV) genotype has been important in understanding HCV classification, epidemiology, evolution, transmission clustering, treatment response, and natural history [4]. According to the latest version of the EASL guideline, the HCV genotype/subtype should be determined with an assay that accurately discriminates HCV subtype 1a from 1b; however, there is no recommendation concerning identification of recombinant HCV strains included [5].

Since the first report about a natural HCV recombinant form described 2002 in Saint Petersburg [6], several recombinant strains have been reported worldwide as a result of recombination between different genotypes and subtypes [7,8]. In Europe, recombination between genotypes 2 and 1 (strain RF1_2k/1b) was found in France, Ireland, Cyprus, Estonia, and Germany and between genotypes 2 and 5 (strain R1) in France [9–15]. In a recent retrospective study, a new HCV genotype test, the cobas^®^ HCV GT (Roche Molecular Systems, Pleasanton, CA, USA), using regions at both ends of the HCV genome was evaluated and results were compared to alternative assays, the TRUGENE^®^ HCV 5´NC Genotyping Kit (Siemens Healthcare Diagnostics Inc., Tarrytown, NY, USA) and the VERSANT^®^ HCV Genotype 2.0 Assay (LiPA) (Siemens) [16]. Among 183 consecutive residual serum samples that had been enrolled in that study in a de-identified manner, four were identified to be possibly infected with a recombinant strain of genotype 2 and subtype 1b. In that study, comparison assays using the 5`end of the virus (core region and/or 5`UTR) for determination of the HCV genotype/subtype revealed HCV subtype 2a and 2a/c, respectively. In contrast, a home-brew NS5B sequencing assay, which uses part of the 3´end of the virus revealed subtype 1b.

The aim of this study was to further analyze these four routine clinical samples. Full-genome sequencing was performed to clarify results and the point of recombination was determined.

## Materials and methods

### Study design

Four anonymized samples collected at baseline that had been enrolled in a study evaluating a new HCV genotyping test, the cobas^®^ HCV GT (Roche), were identified as containing HCV genotype 2 and HCV subtype 1b [16]. These results were compared to those obtained by a commercially available sequencing assay using 5´UTR as target, the TRUGENE^®^ HCV 5´NC Genotyping Kit (Siemens), and a line-probe assay using 5´UTR and core regions, the VERSANT^®^ HCV Genotype 2.0 Assay (LiPA) (Siemens), for which only HCV subtype 2a and subtype 2a/c, respectively, was reported. When the four samples were analyzed with a home-brew sequencing assay using NS5B as target, HCV genotype 1b was found. Based on these results, full-genome next-generation sequencing was performed using a capture based sequencing method and data analyzed using CLC Genomics Workbench to adjudicate the results. Demographic data were collected in a de-identified manner and then linked to the unique HCV sequence.

### Whole genome next-generation sequencing

Nucleic acid purification was performed using the cobas^**®**^ 4800 sample preparation module. Libraries were prepared for Illumina sequencing using the NEBNext^®^ Ultra™ RNA Library Prep Kit for Illumina^®^ (New England Biolabs, Ipswich, MA, USA) according to the manufacturer’s instructions. HCV sequences were captured using the NimbleGen SeqCap EZ probe pool and the NimbleGen SeqCap EZ Library reagent kit according to the manufacturer’s instructions (Roche NimbleGen, Inc., Madison, WI, USA). The capture probe library was custom designed using 117 HCV reference sequences from the Los Alamos HCV Sequence Database (http://hcv.lanl.gov). The pool of 2.1 million probes consists of 29,552 unique oligonucleotides. The libraries were sequenced using the Illumina MiSeq v2 300 cycle kit (Illumina, SanDiego, CA, USA).

The MiSeq reads for each sample were processed via a custom pipeline using CLC Genomics Workbench 8.5.1 (https://www.qiagenbioinformatics.com/). Reads were first mapped to the 117 reference sequence set with all four samples generating strong mappings to complementary regions of Ref.2k.MD.x.VAT96.AB031663 and Ref.1b.CN.x.AY587016.AY587016. A second round of mappings was performed to trimmed versions of the two references. Each sample’s pair of consensus sequences was aligned at the overlap to create sample-specific references for a third mapping iteration, and the final consensus sequences were generated. Average read depth exceeded 1200x and genomic coverage was greater than 99.8% for all samples. All generated full-genome consensus sequences were submitted to the National Center for Biotechnology Information’s (NCBI’s) GenBank (accession nos.: KY780122, KY780123, KY883981, KY883982).

### Identification of the point of recombination and phylogenetic analysis

Phylogenetic, SimPlot and Bootscan analyses were performed on sequences aligned with reference strains representing all HCV genotypes. Data were analyzed with SimPlot v3.5.1 software (http://sray.med.som.jhmi.edu/SCRoftware/) using default settings [17]. Phylogenetic and SimPlot analyses also included nine available 2k/1b full-genome sequences (HQ537006, JX227952, AY587845, HQ537005, FJ821465, KM102765, KM102768, KM102769, KM102770). Two phylogenetic analyses were conducted on the genomic regions to the 5’ end (5'UTR through NS1/p7) and 3’ end (NS3 through NS5B) of the NS2 gene, which was not included. Maximum Likelihood trees were constructed using MEGA 6.0 [18]. The robustness of the relationships in each tree was evaluated by the bootstrap method with 500 replicates.

## Results

In this study, four patients with chronic HCV infection were identified to be infected with the circulating recombinant form (CRF) 2k/1b by use of whole genome next-generation sequencing. Three of four patients were male and the country of origin was found to be Chechnya (n = 2) and Azerbaijan (n = 1). They have been residents in the southeastern part of Austria since 2013, 2014, and 2008 respectively. The fourth patient was a female born in Austria. Three of the four patients including the female had intravenous drug abuse (IVDA) as a risk factor for HCV transmission. Three patients had received antiviral combination therapy with pegylated interferon plus ribavirin prior to introduction of new antiviral regimes with direct-acting antivirals (DAAs). Two of them had shown sustained virologic response (SVR), whereas one patient relapsed. Retreatment with sofosbuvir plus ribavirin was initiated prior to identification of the recombination and resulted in SVR. The fourth patient did not receive any therapy being still HCV RNA positive.

**Fig 1:**
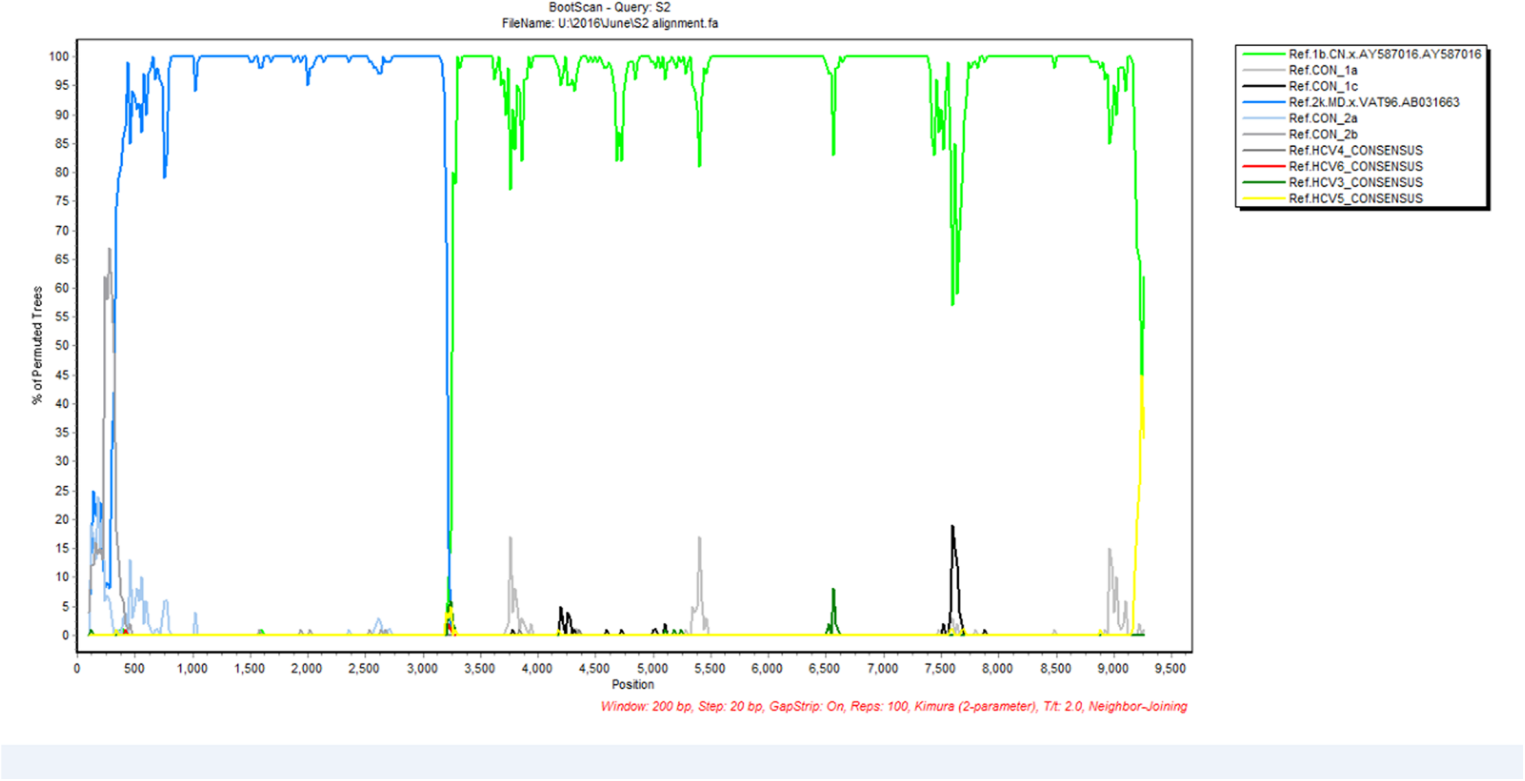
BootScan plot of percentage permuted trees over nucleotide position across the full length of one of the HCV 2k/1b recombinant strains. Different colors represent sequences of different genotypes/subtypes.

**Fig 2:**
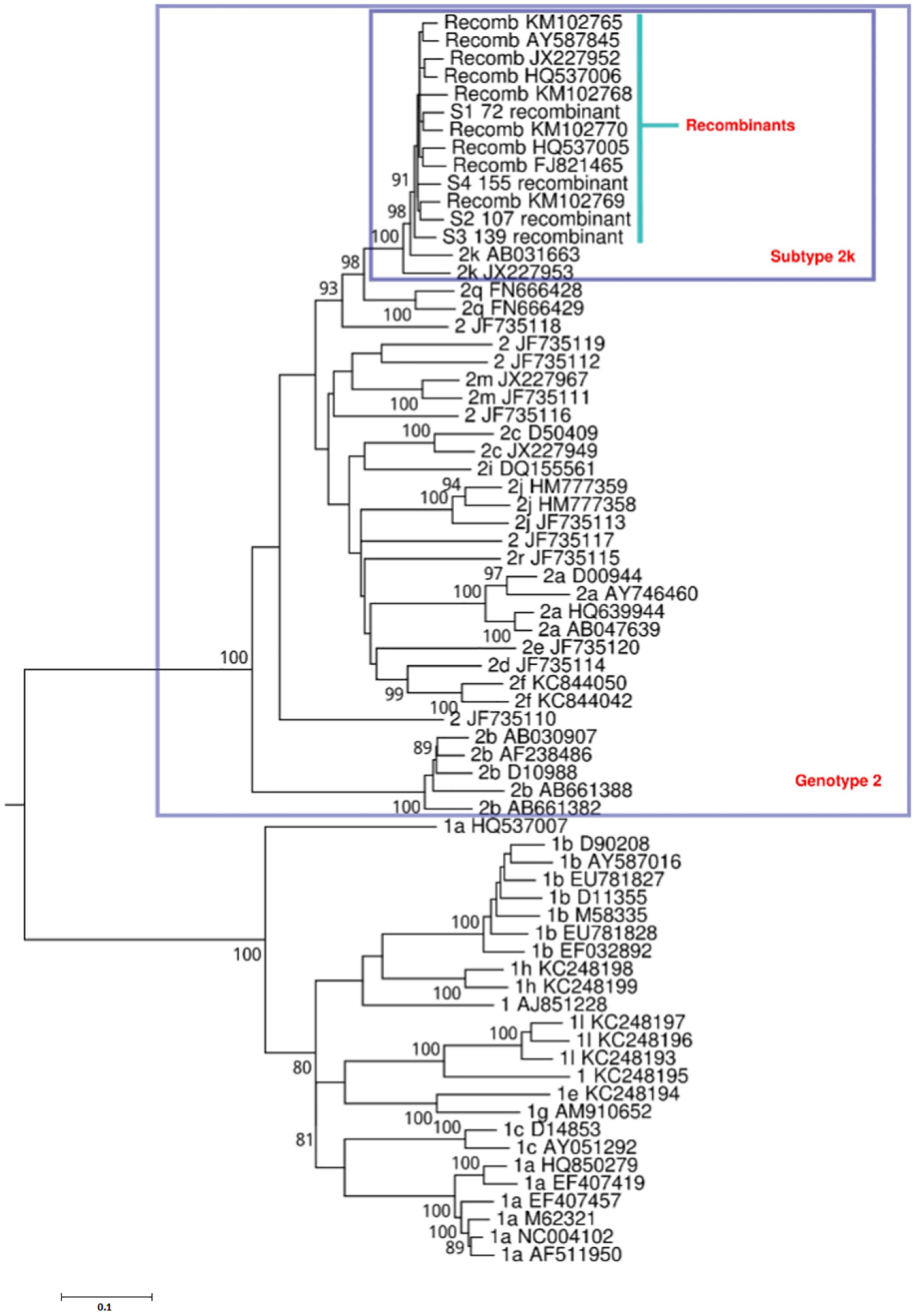
Phylogenetic analysis of the 5'UTR through NS1/p7. Maximum Likelihood tree constructed using MEGA 6.0 using the GTR model of evolution, incorporating assumed rate heterogeneity (Γ, gamma) across sites and invariant sites (I). The four patient samples S1-S4 clustered most closely with genotype 2 and subtype 2k.

**Fig 3:**
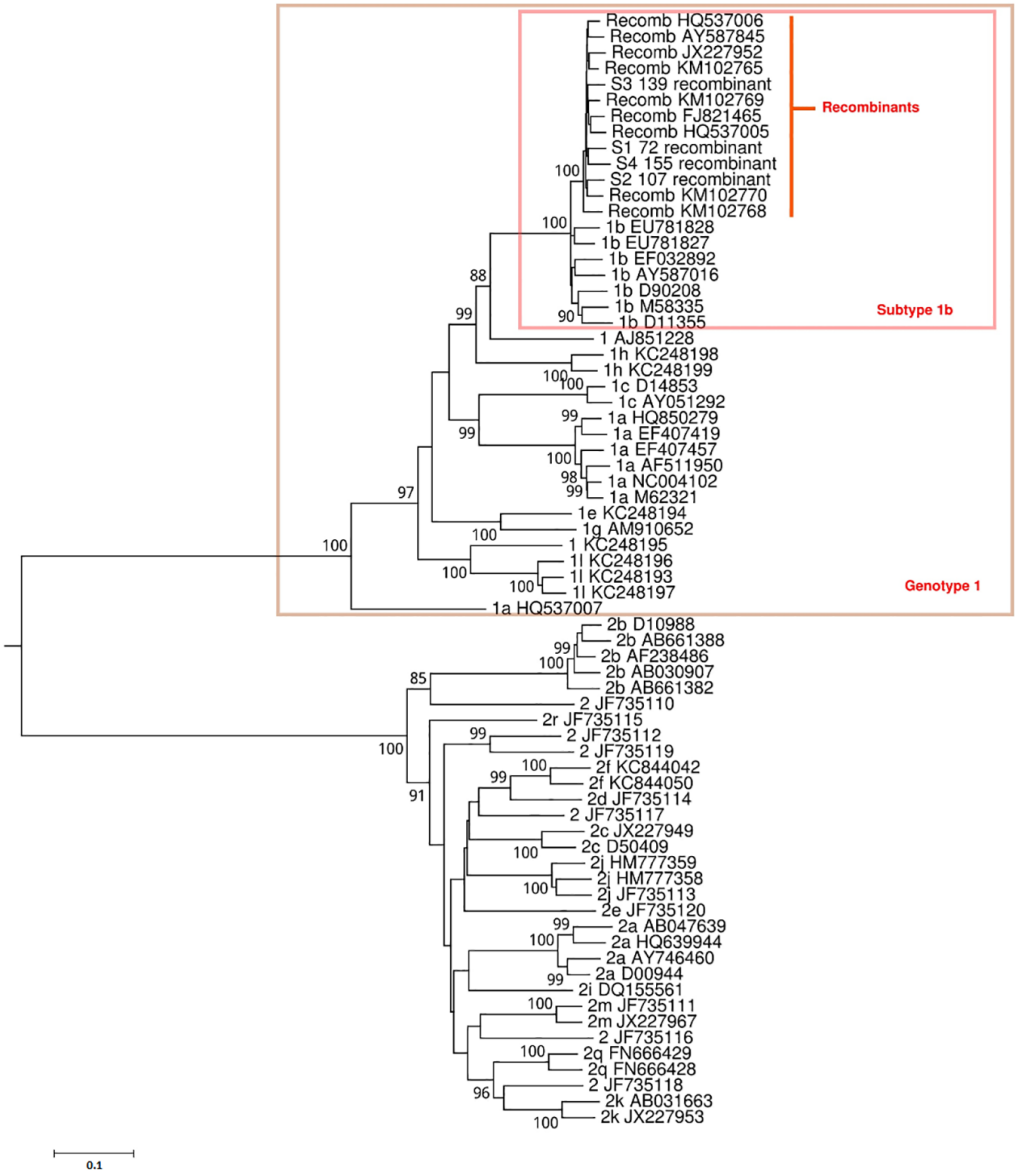
Phylogenetic analysis of the NS3 through NS5B genes. The Maximum Likelihood tree was constructed using MEGA 6.0 using the GTR model of evolution, incorporating assumed rate heterogeneity (Γ, gamma) across sites and invariant sites (I). The four patient samples S1-S4 clustered most closely with genotype 1 and subtype 1b.

Four patient samples collected at baseline had primarily been tested with the TRUGENE^®^ HCV 5´NC Genotyping Kit and classified as genotype 2. Based on the results of the cobas^®^ HCV GT assay, they had been identified to contain HCV genotypes 2 and subtype 1b. Full genome next-generation sequencing using the Illumina instrument identified these viruses as CRF 2k/1b with the point of recombination between nucleotide positions 3189–3200 based on H77 numbering (Fig 1). Phylogenetic analysis showed the four patient sequences formed a monophyletic cluster with CRF1_2k/1b viruses, confirming that these patients were infected with a recombinant HCV (Figs 2 and 3).

## Discussion

Four patients, currently living in the south-eastern part of Austria were identified to be infected with the CRF 2k/1b by use of whole genome next-generation sequencing. The breakpoint was found to be in the HCV NS2 gene consistent with that reported recently [19]. To our knowledge, this is the first report of patients living in Austria being infected with a recombinant HCV strain. The CRF 2k/1b had been detected in patients originating from the Caucasus region and was found to spread over several European countries [6,15,20,21]. In the present study, three of four patients originated from this region (Chechnya and Azerbaijan). It remains unclear whether they had acquired HCV before migration to Austria or later on. However, the patient without migration background may indicate that CRF 2k/1b has already begun to spread over Austria.

The increasing occurrence of recombinant HCV strains may have an impact on the best choice of anti-HCV therapy because several anti-HCV drugs are still non-pangenotypic [5]. For an optimized anti-HCV therapy including patients with recombinant strains, the use of genotyping assays based on a single genomic region or both the 5´UTR and the core region appears to be inappropriate [19]. It is thus recommendable using a genotyping assay including the 5´UTR, Core, and NS5B regions. In the present study, the recombinant HCV strain was indicated by the recently introduced cobas^®^ HCV GT assay. This real-time PCR based assay uses three different target regions in the HCV genome (5’-UTR, Core, and NS5B) to identify HCV genotypes 1 to 6 and genotype 1 subtypes a and b. Identification of more than one HCV genotype/subtype may indicate either mixed infection or presence of a recombinant HCV strain. Confirmation of recombination can be done by use of either population-based or next-generation sequencing. However, next-generation sequencing protocols are currently not useful for the routine diagnostic laboratory because they lack standardization. Furthermore, they are time-consuming and both labor- and cost-intensive.

In conclusion, for identification of the HCV genotype, sequencing techniques appear to be appropriate for identification of HCV recombinant strains; however, these techniques are limited to a few specialized laboratories. Application of a genotyping assay that uses at least two different target regions at both ends of the HCV genome should be employed to identify patients infected with a recombinant HCV strain being of importance regarding the tailored choice of anti-HCV treatment and overall prognosis.
